# Feasibility of Imaging Modalities Combined with a Silicone Gel-Filled Breast Implant in Korean Women

**DOI:** 10.3390/gels9030232

**Published:** 2023-03-16

**Authors:** Pa Hong, Jae Kyoung Kang, Seung Hwan Hwang, Kyung Ah Lee

**Affiliations:** 1 Department of Radiology, Samsung Changwon Hospital, Sunkyunkwan University School of Medicine (SKKU-SOM), Changwon 51353, Republic of Korea; 2 Department of Plastic and Reconstructive Surgery, Jeju National University Hospital, Jeju 63241, Republic of Korea; 3 AVANT Plastic Surgery & Medical Spa, Seoul 06038, Republic of Korea; 4 Department of Plastic and Reconstructive Surgery, Inje University Haeundae Paik Hospital, Busan 48108, Republic of Korea; 5 Korean Academic Association of Aesthetic and Reconstructive Breast Surgery, Seoul 04146, Republic of Korea

**Keywords:** surgical procedures, operative, breast implants, ultrasonography, interdisciplinary

## Abstract

With the occurrence of breast implant crises in Korea, it has become increasingly important to detect complications earlier in patients receiving a device. We have therefore combined imaging modalities with an implant-based augmentation mammaplasty. In this study, we assessed the short-term treatment outcomes and safety of the Motiva Ergonomix^TM^ Round SilkSurface (Establishment Labs Holdings Inc., Alajuela, Costa Rica) in Korean women. A total of 87 women (*n* = 87) were included in the current study. We compared preoperative anthropometric measurements between the right side and the left side of the breast. Moreover, we also compared the thickness of the skin, subcutaneous tissue and the pectoralis major measured on a breast ultrasound preoperatively and 3 months postoperatively. Furthermore, we analyzed the incidences of postoperative complications and the cumulative complication-free survival. Preoperatively, there was a significant difference in the distance from the nipple to the midline between the left and right side of the breast (*p* = 0.000). Both sides of the breast showed significant differences in the thickness of the pectoralis major preoperatively and 3 months postoperatively (*p* = 0.000). A total of 11 cases (12.6%) of postoperative complications occurred; these included five cases (5.7%) of early seroma, two cases (2.3%) of infection, two cases (2.3%) of rippling, one case (1.1%) of hematoma and one case (1.1%) of capsular contracture. Time-to-events were estimated at 386.68 ± 27.79 days (95% CI 334.11–439.27). Here, we describe our experience with imaging modalities in combination with the Motiva Ergonomix^TM^ Round SilkSurface in Korean women.

## 1. Introduction

A silicone gel-filled breast implant (SGBI) is a shell of silicone elastomer that is filled with silicone gel. It is placed either under the breast tissue or the chest muscle of a patient, thus being used for an implant-based augmentation mammaplasty [1]. Its use has been popularized for aesthetic and reconstructive augmentation mammaplasty since the 1960s [2]. Before the emergence of breast implants, developmental abnormalities of the breast were corrected with the implantation of fat tissue or synthetic materials (e.g., sponge) or the injection of silicone or paraffin [3]. Due to concerns over the potential risk of connective tissue disease, however, its use was banned by the US Food and Drug Administration (FDA) [4]. Thus, the US FDA placed a moratorium on the cosmetic use of SGBIs in 1992 due to insufficient long-term safety data [5]. In November 2006, the US FDA approved the clinical use of SGBIs on the condition that their indications were limited to women aged 22 years or older. Then, the US FDA confirmed a lack of a causal relationship between the use of an SGBI and the onset of connective tissue disease or malignancy [6,7].

The prevalent use of an SGBI for aesthetic purposes reflects the growth of the cosmetic industry. This has been recently fueled by advertisement, social media and medical tourism [8]. In particular, medical tourism is a term coined to describe the phenomenon of patients who travel outside their home country in an attempt to receive medical treatments [9]. To date, there has been increasing popularity of aesthetic tourism for several reasons; these include the lower cost, confidentiality, the timely availability of treatment procedures, the recommendations of peers and social media influencers and advertisements [10,11]. Thus, the size of the global market for medical tourism is expected to reach USD 131.35 billion by 2025, with a mean annual increasing rate of 20% [12].

The global market for breast implantation is expected to generate USD 2866.7 million by 2027, growing at compound annual growth rate of 6.55% between 2022 and 2027 [13]. This is closely associated with an increased awareness of the importance of physical appearance, the emergence of an attractive, cohesive SGBI and the diversity of commercially available SGBIs. The size of the global market for breast implants will further increase due to the commercialization of new products and technological advancements [13,14].

Despite the popularity of medical tourism and the expansion of the global market for breast implants, concerns have been raised regarding a lack of standardized regulatory action on cosmetic surgical procedures worldwide. This should be taken seriously because an implant-based augmentation mammaplasty is the most popular cosmetic surgery worldwide [15]. Moreover, it also remains a great concern that medical tourists undergoing cosmetic surgeries are at increased risks of developing postoperative complications [9]. This poses a financial burden to the home healthcare system; Thacoor et al. showed that more than USD 16,000 on average were required for the appropriate management of postoperative complications in each medical tourist receiving cosmetic surgeries. These authors also noted that the possibility of the underestimation of such complications could could not be completely ruled out [9,16]. Specifically, McCrossan et al. reported that medical tourists receiving an implant-based augmentation mammaplasty presented with relatively higher rates of infection (39%) and return to theatre (51%) [9].

Historically, the breast implant industry has been heavily affected by a series of crisis events, thus termed as breast implant crises (BICs) [8]. Global BICs have been classified into the first crisis (Dow Corning), the second crisis (Poly Implant Prothèse) and the third crisis (breast implant-associated anaplastic large cell lymphoma (BIA-ALCL)) [17]. As previously delineated, the Korean breast implant industry has experienced the first crisis (BIA-ALCL) and a second crisis, also known as the first Korean case of a medical device fraud (BellaGel^®^ (HansBiomed Co., Ltd., Seoul, Republic of Korea) breast implant scandal) [18,19,20,21,22,23,24,25]. With the occurrence of the BICs, it has become increasingly important to safeguard patients receiving an SGBI [18].

A wide variety of SGBIs are commercially available for surgery. It is mandatory, however, to select the optimal type of SGBI, which is essential for maximizing the aesthetic outcomes and minimizing the risk of postoperative complications [26]. In this regard, plastic surgeons should consider four major factors affecting the treatment outcomes of an implant-based augmentation mammaplasty; these include (1) patient education and informed consent, (2) tissue-based clinical analysis and planning, (3) refined surgical technique and (4) postoperative regimen. Of these, tissue-based clinical analysis and planning is associated with the choice of the optimal type of SGBI [27]. This can eventually contribute to improving the quality, safety and efficacy of surgery in an evidence-based manner [28].

Over the past 50 years, SGBIs have undergone many changes that are closely associated with their safety, quality and clinical performance [29]. Among such changes, the incorporation of the most advanced silicone technologies into surface texturing have eventually led to the birth of the latest generation of SGBIs [30]. Thus, there have been great changes in the composition of silicone gel as well as the degree of surface texturing of the outer elastomer. This has made it possible for both a plastic surgeon and a patient to choose diverse types of device and surface topography [31].

The surface texturing of an SGBI aims to lower the incidences of common complications of an implant-based augmentation mammaplasty, such as capsular contracture (CC) and the excessive movement of the device in the breast pocket [32,33,34,35]. Although the structure of an SGBI is commonly characterized by a highly cross-linked (cohesive) silicone gel placed in a silicone elastomer shell, there is a unique difference in the process of surface texturing between manufacturers. Three manufacturers of an SGBI, such as Allergan Inc. (Irvine, CA, USA), Mentor Worldwide LLC (Santa Barbara, CA, USA) and Sientra Inc. (Santa Barbara, CA, USA), obtained US FDA approval for the commercial release of their products [36]. In more detail, Allergan Inc. used a lost-salt technique in the manufacturing a textured breast implant, for which the surface was created by dipping a chuck into uncured silicone, which was pressed into a bed of fine, granular salt before drying and then cured in a laminar flow oven. This led to the creation of an irregular surface with pores with a diameter of 600–800 μm and a depth of 150–200 μm [32,37,38]. Mentor Worldwide LLC used negative-contact polyurethane foam to stamp the surface of the device. That is, the chuck was dipped into uncured silicone and the shell was formed accordingly. Then, the uncured silicone shell was pressed into polyurethane foam to imprint pores with a diameter of 70–150 μm and a height of 40–100 μm. Mentor Worldwide LLC has manufactured a round breast implant with approximately 100 pores/inch and a shaped device with 65 pores/inch [32,37,38]. For proprietary reasons, Sientra Inc. has not revealed its texturing process.

The use of a textured breast implant has been advocated based on the argument that it is useful in lowering the rates of malposition, decreasing the risk of CC and providing superior cosmetic outcomes compared with a smooth device [26,29,33,39,40,41]. Unlike a smooth breast implant, a textured device is advantageous in forming an anatomical shape, also known as a teardrop shape. Presumably, this might produce more natural outcomes of an implant-based augmentation mammaplasty [42,43]. Still, however, there is a paucity of data supporting the scientific evidence of the advantages of a textured breast implant over a smooth device. Some early studies have shown lower rates of CC in patients receiving a textured breast implant compared with those receiving a smooth device. However, this has been contradicted by other studies showing similar rates between the two devices [44,45,46,47,48].

The emergence of a textured breast implant was followed by the development of a microtextured device that is characterized by a surface with a miniaturized roughness [49]. Indeed, commercially-available SGBIs are equipped with surface topographies, such as smooth, microtextured and macrotextured surfaces [50].

The Motiva Ergonomix^TM^ Round SilkSurface (Establishment Labs Holdings Inc., Alajuela, Costa Rica) is the latest generation of an SGBI with a microtextured surface. It is closely associated with the popularity of microtextured devices in the Korean market. Its 3-year safety has been recently assessed using high-resolution ultrasound (HRUS) [24]. Indeed, the importance of the use of HRUS in assessing the safety of SGBIs has been well documented in the literature [18,19,20,21,22,23,24,25,51,52]. Along the continuum of these previous studies, we have efficiently used three-dimensional (3-D) simulation technology and HRUS to maximize both the aesthetic outcomes and safety of the Motiva Ergonomix^TM^ Round SilkSurface. Here, we describe plastic surgeons’ experience with imaging modalities in combination with the Motiva Ergonomix^TM^ Round SilkSurface in Korean women. Its safety in a cohort of Korean women has been well documented [21,24,25,53].

## 2. Results and Discussion

### 2.1. Demographic and Clinical Characteristics of the Patients

A total of 87 women (*n* = 87; mean age = 33.79 ± 7.68 years old and mean follow-up period = 183.14 ± 158.03 days) were included in the current study. Their demographic and clinical characteristics are summarized in Table 1.

### 2.2. Differences in the Anthropometric Measurements between the Left and Right Side of the Breast

Differences in the anthropometric measurements between the left and right side of the breast are summarized in Table 2. This showed a significant difference in the distance from the nipple to the midline between the left and right side of the breast (9.26 ± 1.01 vs. 8.46 ± 0.86 cm, *t* = 4.841, *p* = 0.000). This indicates that the patients with a significant difference in the distance from the nipple to the midline between the two sides of the breast should be corrected for symmetry. A preoperative assessment of breast anthropometrics is an essential factor for breast surgery [54]. This enables plastic surgeons to predict the volume of resection or implantation based on practical and reproducible data in the preoperative work-up [55]. A preoperative simulation of the postoperative outcomes is therefore mandatory to achieve bilateral symmetry [56]. It also helps to achieve an aesthetically balanced profile with the guidance of breast measurement data [57].

### 2.3. Time-Dependent Changes in the Thickness of the Dermis, Subcutaneous Tissue and Pectoralis Major Measured on HRUS

**Figure 1:**
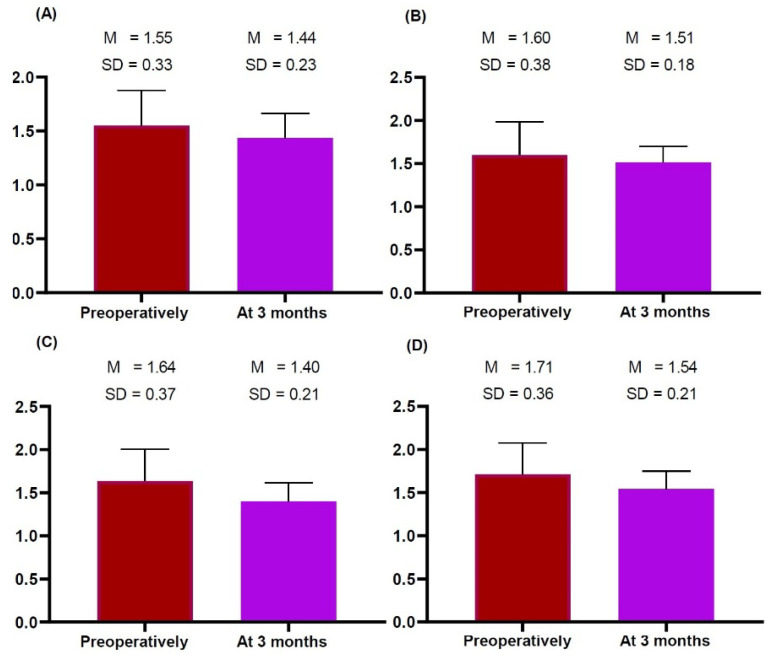
Differences in the thickness of the dermis measured on breast ultrasound preoperatively and 3 months postoperatively. Preoperatively and 3 months postoperatively, the thickness of skin was measured (**A**) in the right superior, (**B**) the right inferior, (**C**) the left superior and (**D**) the left inferior region of the breast. This showed no significant differences in the thickness of skin between the preoperative and 3-months postoperative measurements (*p* > 0.05). Note: The *y*-axis indicates measurements.

**Figure 2:**
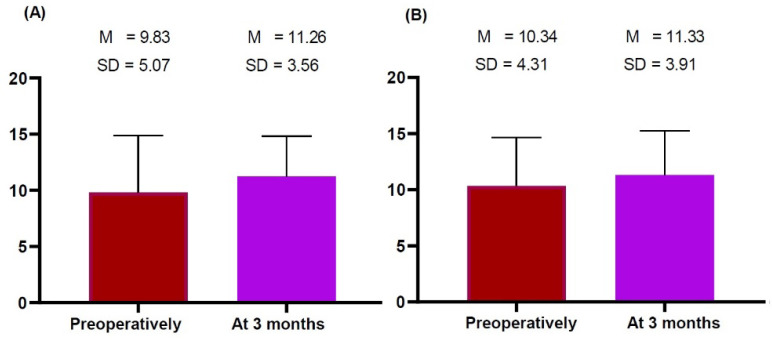
(**A**,**B**) Differences in the thickness of subcutaneous tissue measured on breast ultrasound preoperatively and 3 months postoperatively. Preoperatively and 3 months postoperatively, the thickness of subcutaneous tissue was measured on both sides of the breast. This showed no significant differences in the thickness of subcutaneous tissue between the preoperative and 3-months postoperative measurements (*p* > 0.05). Note: The *y*-axis indicates measurements.

**Figure 3:**
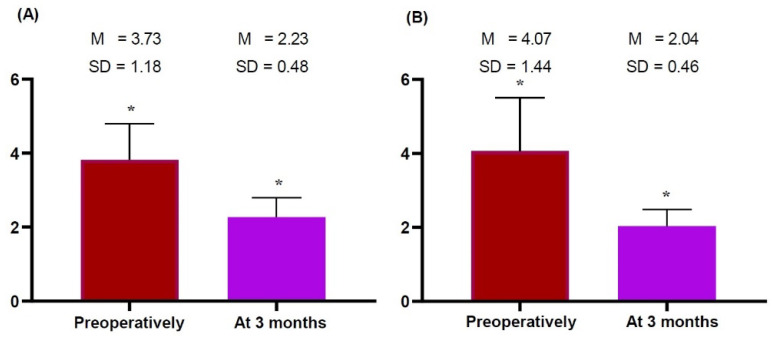
(**A**,**B**) Differences in the thickness of the pectoralis major measured on breast ultrasound preoperatively and 3 months postoperatively. Preoperatively and 3 months postoperatively, the thickness of pectoralis major was measured on both sides of the breast. This showed significant differences in the thickness of subcutaneous tissue between the preoperative and 3-months postoperative measurements (right side: 3.73 ± 1.18 vs. 2.23 ± 0.48 mm, *t* = 5.633, *p* = 0.000 and left side: 4.07 ± 1.44 vs. 2.04 ± 0.46 mm, *t* = 4.882, *p* = 0.000). Note: The *y*-axis indicates measurements. * Statistical significance at *p* < 0.05.

We obtained measurements of the thickness of the dermis, subcutaneous tissue and pectoralis major preoperatively and at 3 months postoperatively, as summarized in Table 3 and Figure 1, Figure 2 and Figure 3.

Both sides of the breast showed no significant differences in the thickness of the dermis and subcutaneous tissue preoperatively and 3 months postoperatively (Table 3; Figure 1 and Figure 2, respectively). However, they showed significant differences in the thickness of the pectoralis major preoperatively and 3 months postoperatively (right side: 3.73 ± 1.18 vs. 2.23 ± 0.48 mm, *t* = 5.633, *p* = 0.000 and left side: 4.07 ± 1.44 vs. 2.04 ± 0.46 mm, *t* = 4.882, *p* = 0.000) (Figure 3). This indicates that patients receiving a breast implant are vulnerable to an increase in the thickness of the pectoralis major 3 months postoperatively.

### 2.4. Aesthetic Outcomes

**Figure 4:**
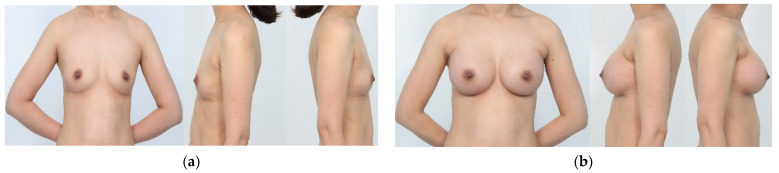
Illustrative case. A 38-year-old woman received the Motiva Ergonomix^TM^ Round SilkSurface (ERSF, 335 cc) for both sides of the breast. The patient was satisfied with the aesthetic outcomes ((**a**): preoperatively and (**b**): 3 months postoperatively).

An illustrative case is shown in Figure 4.

### 2.5. Safety Outcomes

A total of 11 cases (12.6%) of postoperative complications occurred; these included five cases (5.7%) of early seroma, two cases (2.3%) of infection, two cases (2.3%) of rippling, one case (1.1%) of hematoma and one case (1.1%) of CC (Table 4). The patients presenting with early seroma, infection, rippling, hematoma and CC were treated using aspiration, explantation, replacement with other devices, evacuation and revision, respectively.

**Figure 5:**
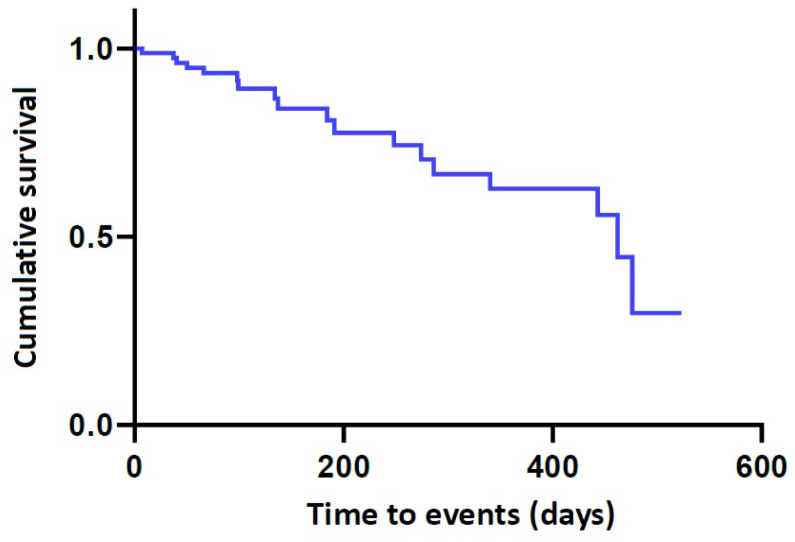
Kaplan–Meier cumulative survival. In our series, the time-to-events were estimated at 386.68 ± 27.79 days (95% CI 334.11–439.27).

The time-to-events (TTEs) were estimated at 386.68 ± 27.79 days (95% CI 334.11–439.27) (Table 5). The corresponding Kaplan–Meier cumulative survival was plotted as a curve (Figure 5).

With the identification of a causal relationship between a textured breast implant and the onset of BIA-ALCL, there has been controversy surrounding the use of a textured device [58]. This is supported by the suggestion that a textured breast implant should no longer be used because of its association with a risk of BIA-ALCL [59]. Although the US FDA did not recommend that asymptomatic patients receiving a textured breast implant undergo explantation, there is still concern regarding the risk of BIA-ALCL and such patients are in need of guidance as to the risks and benefits of the replacement of a textured device with a smooth one [59,60]. The US FDA finally requested the immediate withdrawal of BIOCELL breast implants and tissue expanders (Allergan Inc.) from the market on 24 July 2019 and the manufacturer issued a global recall of products [61].

Controversial opinions exist regarding the discontinued use of textured breast implants. Efforts have been made to reduce the risk of BIA-ALCL, for which the use of a textured breast implant has shifted to that of a smooth device among plastic surgeons [62,63]. The use of textured breast implants was banned in Korea on 29 August 2019, as mandated by the KMFDS, after it was reported that three cases of BIA-ALCL occurred in Korea between 2019 and 2020 (16 August, 24 December 2019 and 5 October 2020) [24]. The Korean market for SGBIs has been characterized by the popularity of microtextured devices since the Motiva Ergonomix^TM^ Round SilkSurface was approved by the KMFDS on 17 June 2016. The shift from textured breast implants to microtextured devices is an interesting phenomenon in Korea [24]. According to Weltz et al., the risks of developing BIA-ALCL might be highest with textured breast implants, followed by microtextured and smooth devices in decreasing order [64].

Despite the advancement of breast implant technology and surgical techniques, there have been no changes in the occurrence of complications of implant-based augmentation mammaplasty. Among such complications, CC and the rupture of a breast implant remain serious events. It is therefore mandatory to perform continuous monitoring of the possible complications of an implant-based augmentation mammaplasty, which is essential for ensuring the safety of patients receiving a device [30].

The Motiva Ergonomix^TM^ Round SilkSurface is an innovative type of a product whose characteristics are distinguishable from those of other manufacturers. That is, its visible barrier layer, nanoscale smooth surface and optional radiofrequency are advantageous in ensuring postoperative safety [65]. Moreover, its surface properties are closely associated with decreased incidences of complications, such as CC, thus making it efficient in lowering the frequency of reoperation to <1% [66]. Indeed, there were no cases of CC of Baker grade III/IV according to a single-center study conducted in patients undergoing augmentation mammaplasty using Motiva implants. Thus, a risk of chronic inflammation is minimized [66,67].

The size, shape and projection of breast implants have been diversified to cater for the needs of patients who are in need of augmentation mammaplasties by preserving the natural appearance of the breast, which poses a challenge for plastic surgeons. Round breast implants were formerly used to improve the upper pole fullness, but anatomical ones have become available to maximally imitate the natural shape of the breast by providing more fullness in the lower pole [68]. Despite the proven effectiveness and safety of anatomical implants, their disadvantages include the requirement of an advanced level of surgical technique as well as increased risks of malrotation, whose incidence reaches up to 5.2% [37]. In this context, the Motiva Ergonomix^TM^ Round SilkSurface is useful in fulfilling two goals: a natural appearance and feel of the breast and a reduction in the risks of malrotation. Thus, it has efficiently combined its rheologic properties with the use of a specific elastomer shell, thus adjusting with gravity to the patient’s position, as previously described [69].

Currently in Korea, diverse types of SGBI are commercially available, and their safety profile varies according to the manufacturer. It would therefore be mandatory to consider the safety profile of each device when selecting the optimal type of device for Korean women who are in need of an implant-based augmentation mammaplasty [14]. Indeed, approximately 77,000 SGBIs were annually used for surgery in the Korean market between 2016 and 2020. There were notable changes in the Korean market between January and June of 2021 after the occurrence of the first Korean case of medical device fraud. That is, the product sales by manufacturer during this period were as follows: Mentor Worldwide LLC. (*n* = 15,570), Establishment Labs Holdings Inc. (*n* = 9732), Groupe Sebbin SAS (*n* = 7374), GC Aesthetics PLC (*n* = 1406), Allergan Inc. (*n* = 145) and Silimed Inc. (*n* = 2) in decreasing order [23]. Indeed, the Motiva Ergonomix^TM^ Round SilkSurface might be the device of choice for Korean women who have been faced with the BIA-ALCL crisis and the first Korean case of medical device fraud [24,70]. The cost of the Motiva Ergonomix^TM^ Round SilkSurface is the highest, although it triggered a boom in microtextured devices [71]. As described above, 15,570 and 9732 devices were sold by Mentor Worldwide LLC. and Establishment Labs Holdings Inc., respectively, between January and June of 2021 [23]. Considering that their costs are USD 5070.01 and 8450.02 in corresponding order, it can be inferred that the total revenue of their sales reached USD 78,940,055.7 and 82,235,594.64 [71]. This indicates that the Motiva Ergonomix^TM^ Round SilkSurface is the most popular brand of SGBI in Korea. A recent study also reported that breast cancer survivors receiving the Motiva Ergonomix^TM^ Round SilkSurface achieved improvements in quality of life following a 4-week nurse-led exercise rehabilitation [72].

To date, evidence-based efforts have been made to define the safety of the Motiva Ergonomix^TM^ Round SilkSurface in Korea. Previous studies have shown that patients receiving the Motiva Ergonomix^TM^ Round SilkSurface presented with postoperative complications at rates of 6.58–12.82% [21,24,25,53,71,73]. This is in agreement with our results showing that there were a total of 11 cases (12.6%) of postoperative complications (five cases (5.7%) of early seroma, two cases (2.3%) of infection, two cases (2.3%) of rippling, one case (1.1%) of hematoma and one case (1.1%) of CC). As shown in the current results, however, the highest incidence (5.7%) of early seroma remains problematic. According to Sforza et al., body mass index (BMI) > 30 kg/m^2^, the volume of breast implant > 350 cc, the submammary pocket and a smoking habit served as risk factors of early seroma [74]. Considering that our clinical series of patients had a mean BMI of 20.38 ± 1.16 kg/m^2^, however, we assume that the high incidence of early seroma might arise from foreign body reactions, as previously advocated [75].

Global researchers have also evaluated the safety of the Motiva Ergonomix^TM^ Round SilkSurface [66,76,77,78,79,80,81,82,83,84]. These efforts have focused on the effects of its surface property on the occurrence of CC [49,66,76,77,78,79,80,81,82,83,84]. One of these efforts deserves particular mention [84]. To date, contradictory opinions have existed regarding whether there is a difference in the risk of CC between breast implants with varying surface topographies [85,86]. However, this was refuted by Doloff et al., who provided experimental evidence showing that there were differences in immune responses depending on the surface topography of an SGBI [84].

The results of the current study cannot be generalized. First, we failed to consider the prospective design. Prospective studies are more reliable in providing more scientifically reliable results compared with retrospective ones [87]. Second, we failed to analyze the mechanical behavior of the Motiva Ergonomix^TM^ Round SilkSurface. This deserves further studies based on mathematical and mechanical models, as proposed by the existing literature [88,89].

## 3. Conclusions

Here, we describe our experience with imaging modalities, including HRUS, in combination with an implant-based augmentation mammaplasty using the Motiva Ergonomix^TM^ Round SilkSurface in Korean women. This deserves further large-scale, prospective studies.

## 4. Patients and Methods

### 4.1. Study Design

Following the occurrence of the first Korean case of medical device fraud, a total of 87 Korean women (174 breasts) received the Motiva Ergonomix^TM^ Round SilkSurface for aesthetic purposes at our hospitals between December 2020 and November 2022 [24,25]. We included women aged 18 years or older with normal physical development. However, we excluded patients with factors that may have affected the measurements of the anthropometric parameters (e.g., endocrine disorder, poor systemic health conditions and a past history of breast surgery). The current study followed the applicable laws, regulations and ethics guidelines. The patients submitted written informed consent for the use of their preoperative and postoperative data and findings for publication of this article.

### 4.2. Combination of an Implant-Based Augmentation Mammaplasty with Imaging Modalities

**Figure 6:**
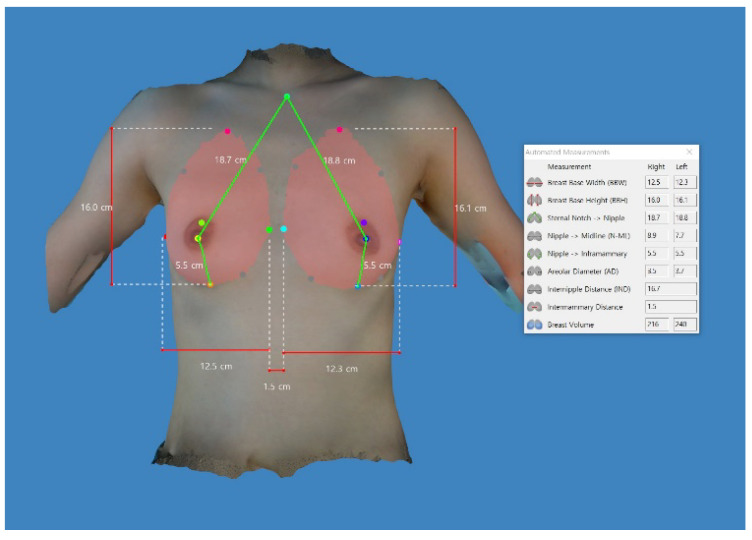
Preoperative simulation of the postoperative outcomes using the Divina^TM^ 3-dimensional scanner. The anthropometric measurements are preoperatively obtained; these include breast base width, breast base height, distance from the sternal notch to the nipple, distance from the nipple to the midline, distance from the nipple to the inframammary fold, breast volume, internipple distance and intermammary distance.

Preoperatively, we simulated the postoperative outcomes using the Divina^TM^ 3-D scanner (Establishment Labs Holdings Inc.) by measuring breast anthropometrics (Figure 6) [24,70].

Previous studies have shown that the suppression of bacterial colonization is effective in lowering the risk of CC to <1% [90,91]. It has also been documented that risks of CC or BIA-ALCL might be associated with bacterial infection [92,93,94,95].

Surgery was performed after anesthetic induction with the prophylactic use of intravenous antibiotics (Cefazolin 1 g; Yuhan Corporation, Seoul, Republic of Korea). Under general anesthesia or intravenous sedation, all surgical procedures were performed in accordance with a 14-point plan, as previously proposed [96,97,98]. We also considered that peri-areolar or transaxillary incisions are associated with a higher incidence of CC [99,100]. We suppressed the bacterial entry into the pocket using nipple shields. We avoided using a subglandular pocket. We also avoided performing dissections into the breast parenchyma while minimizing devascularized tissue and performing hemostasis. We irrigated the pocket using Betadine Triple Antibiotic (Betadine-Triple) (50 cc Betadine solution, 50,000 units bacitracin, 1 g cefazolin, 80 mg gentamycin, 500 cc normal saline), 50–50% Betadine solution and normal saline or a non-Betadine containing triple antibiotic solution (NB-TAB) (50,000 units bacitracin, 1 gm cefazolin, 80 mg gentamicin, 500 cc NS) [101,102]. Finally, we placed the Motiva Ergonomix^TM^ Round SilkSurface in a pocket [53]. 

We reduced the risk of skin contamination using a wipe/prep skin, barrier or sleeve. We shortened the implant open time and replaced sizers. While changing surgical gloves prior to handling, we used new or cleaned instruments and drapes. However, we did not use a drainage tube because it may be a potential site of bacterial entry [103]. We closed incisions using layered sutures in the breast tissue while using skin adhesive or surgical tape to close the skin. Finally, we covered subsequent procedures that may breach the skin or mucosa using prophylactic antibiotics [53,104].

The patients underwent stringent postoperative monitoring at 1, 2, 3, and 4 weeks; 3, 6, 9, and 12 months; and thereafter using HRUS (Aplio i600; Canon Medical System, Otawara, Tochigi, Japan) [14,18,21,22,23,24]. 

To assess the possible occurrence of postoperative swelling, we measured the thickness of the dermis, subcutaneous tissue and pectoralis major on HRUS. Then, we compared measurements preoperatively and 1 and 3 months postoperatively, as previously described [24,70].

### 4.3. Aessment Criteria

We compared anthropometric measurements, such as breast base width, breast base height, distance from the sternal notch to the nipple, distance from the nipple to the midline, distance from the nipple to the inframammary fold (IMF), areolar diameter, breast volume, internipple distance and intermammary distance, between the left and right side of the breast. Moreover, we also monitored time-dependent changes in the thickness of the dermis, subcutaneous tissue and pectoralis major muscle preoperatively and 1 and 3 months postoperatively [24,70]. Furthermore, we analyzed incidences of postoperative complications, as previously described [21,24,25,53,71,73]. We also estimated the complication-free survival rates of the Motiva Ergonomix^TM^ Round SilkSurface, as previously described [21,24,25,53,71,73].

### 4.4. Data Analysis

Measurements are expressed as mean ± standard deviation or the number of the patients with percentage. The differences in measurements between the left and right side of the breast, or preoperatively and postoperatively 1 and 3 months were analyzed using the Student’s *t*-test. To analyze the survival rates of the Motiva Ergonomix^TM^ Round SilkSurface, we estimated the TTEs, defined as the length of time until a well-defined end point of interest occurs, expressed as the percentage of the Motiva Ergonomix^TM^ Round SilkSurface remaining without undergoing revision or removal. Then, we plotted the Kaplan–Meier complication-free survival curve, for which the cumulative overall complication-free survival was estimated, and 95% confidence intervals (CIs) were provided [14,21,24,25,53,71,73,105]. Statistical significance was accepted as *p* < 0.05.

## Figures and Tables

**Table 1:** Demographic and clinical characteristics of the patients (*n* = 87).

Variables	Values
Age (years old)	33.79 ± 7.68
Sex (male-to-female ratio)	0:87
BMI (kg/m^2^)	20.38 ± 1.16
FU period (days)	183.14 ± 158.03
Purpose of surgery	
Aesthetic augmentation mammaplasty	86 (94.3%)
Type of incision	
Axillary incision	74 (85.1%)
IMF incision	7 (8.0%)
Peri-areolar incision	6 (6.9%)
Volume of breast implant	
≤245	9 (10.4%)
250–295	26 (29.9%)
300–345	23 (26.4%)
350–395	16 (18.4%)
≥400	13 (14.9%)

Abbreviations: BMI, body mass index; FU, follow-up; IMF, inframammary fold. Values are presented as mean ± standard deviation or the number of cases with percentage.

**Table 2:** Preoperative anthropometric measurements obtained on the Divina^TM^ 3-dimensional scanner.

Variables	Values		*t*	*p*-Value
	Right	Left		
Breast base width	12.80 ± 1.06	12.97 ± 1.10	−1.298	0.200
Breast base height	15.84 ± 1.22	15.94 ± 1.32	−1.440	0.156
Distance from the sternal notch to the nipple	18.23 ± 1.42	18.13 ± 1.48	1.234	0.223
Distance from the nipple to the midline	9.26 ± 1.01	8.46 ± 0.86	4.841	0.000 *
Distance from the nipple to the inframammary fold	5.48 ± 0.74	5.48 ± 0.90	−0.025	0.980
Breast volume	189.67 ± 64.83	207.29 ± 66.57	−2.896	0.006 *
Internipple distance	17.84 ± 1.54		Non-applicable	
Intermammary distance	2.10 ± 0.59		Non-applicable	

Values are presented as mean ± standard deviation. * Statistical significance at *p* < 0.05.

**Table 3:** The thickness of the dermis, subcutaneous tissue and pectoralis major measured on breast ultrasound.

Variables	Values		*t*	*p*-Value
	Preoperatively	3 Months Postoperatively		
Skin				
Right superior	1.55 ± 0.33	1.44 ± 0.23	1.252	0.233
Right inferior	1.60 ± 0.38	1.51 ± 0.28	0.863	0.404
Left superior	1.64 ± 0.37	1.40 ± 0.21	1.808	0.094
Left inferior	1.71 ± 0.36	1.54 ± 0.21	1.600	0.134
Subcutaneous tissue				
Right	9.83 ± 5.07	11.26 ± 3.56	−1.609	0.128
Left	10.34 ± 4.31	11.33 ± 3.91	−1.108	0.285
Pectoralis major				
Right	3.73 ± 1.18	2.23 ± 0.48	5.633	0.000 *
Left	4.07 ± 1.44	2.04 ± 0.46	4.882	0.000 *

Values are presented as mean ± standard deviation. * Statistical significance at *p* < 0.05.

**Table 4:** Postoperative complications.

Variable	Value
Early seroma	5 (5.7%)
Infection	2 (2.3%)
Rippling	2 (2.3%)
Hematoma	1 (1.1%)
CC	1 (1.1%)

Abbreviations: CC, capsular contracture. Values are presented as the number of the patients with percentage.

**Table 5:** Overall complication-free survival.

N	n	Censored Value	Time-to-Events (months)	95% CI
87	18	69 (79.3%)	386.68 ± 27.79	334.11–439.27

Note: N, total number of cases; n, incidences of postoperative complications; CI, confidence intervals. Values are presented as mean ± standard error or the number of patients with percentage, where appropriate.

## Data Availability

The data presented in this study are available on request from the corresponding author. The data are not publicly available for privacy reasons.
